# The Juxtaoral Organ: From Anatomy to Clinical Relevance

**DOI:** 10.3390/diagnostics12020552

**Published:** 2022-02-21

**Authors:** Gaia Favero, Rita Rezzani, Luigi Fabrizio Rodella

**Affiliations:** 1 Anatomy and Physiopathology Division, Department of Clinical and Experimental Sciences, University of Brescia, 25123 Brescia, Italy; rita.rezzani@unibs.it (R.R.); luigi.rodella@unibs.it (L.F.R.); 2 Interdipartimental University Center of Research “Adaptation and Regeneration of Tissues and Organs (ARTO)”, University of Brescia, 25123 Brescia, Italy

**Keywords:** juxtaoral organ, anatomy, clinical pitfall

## Abstract

The juxtaoral organ was first described 1885 as a rudimentary structure that developed and disappeared in the embryonic period. Since then, it has been studied further and is now known to be a permanent anatomical structure of considerable importance in clinical, surgical and pathological fields. However, there are no precise and uniform descriptions about its anatomical localization and functional significance. Precise and in-depth anatomical knowledge is crucial to reducing the risk of incorrect identification of the juxtaoral organ, due to fact that this anatomical structure can be misinterpreted as a carcinoma, leading to unnecessary treatments. Therefore, the purpose of this review is to summarize the actual knowledge on the gross and microscopic anatomy of the juxtaoral organ and outline its clinical relevance in order to prevent unnecessary investigations/treatments of this anatomical pitfall. We believe that further studies are still needed to add new perspectives in relation to the juxtaoral organ.

## 1. Introduction

The ‘Chievitz Organ’ is a neuroepithelial (primarily) bilateral anatomical structure located laterally to the walls of the oral cavity and featuring circumscribed epithelial cell nests [1,2,3,4,5].

In 1885, during studies on salivary gland organogenesis in 10-week-old embryos, the Danish histologist Johan Henrik Chievitz first described this anatomical structure (from whom the name is derived) and presented it as a vestigial organ that disappeared before birth [6]. In 1953, Wolfgang Zenker and his research group proved that the organ of Chievitz is a mass of nerve fibres and sensory receptors containing epithelial cells, located within the soft tissue between the medial surface of the mandible and the buccinator muscle and that it presents abundant innervation from the buccal nerve. Notably, Zenker found that the organ of Chievitz persists in adults without involution during the physio pathological process of aging [7,8]. Due to its topography, Salzer and Zenker proposed defining this anatomical structure as the ‘juxtaoral organ’, as it was later recognized in Terminologia Histologica: International Terms for Human Cytology and Histology (FICAT, 2008) and in the International Anatomical Terminology [9]. To date, this organ may be found with various other names, such as orbital inclusion, buccopharyngeal tract, ramus mandibularis ductus parotidei or buccotemporal organ [10]. Furthermore, Zenker speculated that the juxtaoral organ, due to its close relation with the buccal nerve, might have a mechanosensory function [11]. Subsequently, various authors have suggested that the juxtaoral organ might be a mechanoreceptor organ involved in different sensory information concerning the lateral wall of the oral cavity, such as sucking, deglutition, mastication and the protection of wall reflexes and tonus [12,13,14]. Recently, Suárez-Quintanilla et al. [15] provided additional indirect findings, through analyses of the innervation and the expression of putative mechanoproteins required for mechanosensing and mechanotransduction, which might confirm its mechanoreceptor function, at least during development. It was also hypothesized that the juxtaoral organ might become differentiated during development and thus might perform both secretory activity and neuroreceptor or sensory functions. Despite the various studies on the juxtaoral organ, to date, the possible function(s) of the juxtaoral organ remains speculative.

According to Zenker and his research group, the juxtaoral organ first appears in embryos of 0.75–1.2 cm in length in the oral cavity and it has been hypothesized that it may originate from the closely associated buccal nerve [16]. In particular, the juxtaoral organ develops from the ectoderm of the lateral wall of the oral cavity as a tubule at the sulcus buccalis that invaginates into the mesenchyme. However, during prenatal development, the connection with the oral cavity disappears [15]. Mérida-Velasco et al. [17], through the evaluation of 55 human embryos and 95 foetuses at different stages of development, described the morphogenesis of the juxtaoral organ in humans and, interestingly, reported that it comes from the epithelium of the lower segment of the transverse opening of the stomodeum during O’Rahilly Stage 16, subsequently separating from the epithelium in O’Rahilly Stage 18. After week 11, the authors observed the formation of the connective tissue capsule.

The human embryological development of the juxtaoral organ was summarized by D’Andrea and his colleagues [1,12], as follows:

i. The oral part, which descends caudally to the site where the parotid duct penetrates the buccinator muscle. Due to this part disappearing during embryological development, some authors reached the incorrect conclusion that the juxtaoral organ may be a rudimentary organ;
ii. The middle part, which crosses the buccal nerve;
iii. The dorsal part, which is correlated to the oral part of the medial pterygoid muscle.

Progressively, the epithelial components of juxtaoral organ are surrounded by the mesenchymal components of the organ.

Furthermore, because of the genetic kinship between the juxtaoral organ and the parotid gland, some authors suggested that the juxtaoral organ may be an elongation of the parotid gland. To date, no complete and validated links have been observed between the juxtaoral organ and the parotid gland epithelium and, additionally, the juxtaoral organ appears earlier than the parotid gland: the juxtaoral organ is observed in embryos of 0.75–1.2 cm in length, whereas the parotid gland occurs in embryos of 1.6–2 cm in length [4].

The juxtaoral organ is phylogenetically preserved and, notably, is present in a wide variety of species, including mammals (not only in humans), birds, fish, amphibians and reptiles [8,16]. This observation suggests that the juxtaoral organ could be relevant in embryological process(es) related to evolutionary development. Although, to date, the juxtaoral organ has been studied more often in humans as compared to other species, Ito and colleagues [18] have reported and meticulously described the organogenesis, morphology and anatomy of the juxtaoral organ in mice. The authors found that the juxtaoral organ in mice presents some important differences as compared to that found in humans. At embryonic day 12, it was observed that the juxtaoral organ in mice appeared as an epithelial cord at the commissura buccalis and then separates from it soon after, moving to the submucosal space. Thereafter, the anterior and posterior ends of the juxtaoral organ cord extend anteriorly to the lateral fascia of the masseter muscle on the mandible and posteriorly to the submucosal space of the soft palate. Through electron microscopical evaluation, Ito et al. [18] also reported that the parenchyma of the juxtaoral organ in mice presents an epithelial cord of non-keratinized cuboidal cells surrounded by a basal membrane and connective tissue; however, no secretion vesicles are observed among the epithelial cells of the juxtaoral organ. This reported absence of secretion vesicles in the juxtaoral organ in mice suggests that the juxtaoral organ may not exhibit endocrine or exocrine properties and that it cannot even be considered partially contractile. The study by Ito and colleagues [18] also suggested that the differences observed are linked to the phylogenetic order and the functional requirements of different species and stressed of the need for further studies on comparative anatomy and embryogenesis in other species to support/contradict their hypotheses. Kobayashi et al. [19] studied the development of the juxtaoral organ in mice and confirmed that its morphology is very different from that in humans. Notably, for the first time, the authors described the immunohistochemical and ultrastructural characteristics of the epithelial, mesenchymal and neuronal components of the juxtaoral organ in both developing embryos and mature mice. They reported that the juxtaoral organ in mice is adjacent to the fascia of the masticatory muscles during all the developmental stages examined. Especially in the middle and posterior segments, the juxtaoral organ is outside the buccinator muscle, but remains located adjacent to the oral mucosal epithelium. Furthermore, Kobayashi and colleagues [19] observed in 8-week-old mice, that the parenchyma of the juxtaoral organ is positive for p63, suggesting that the cell cycle of the parenchymal cells in the juxtaoral organ of adult mice is stopped or proceeds very slowly, unlike the rapid turnover of the oral epithelial cells. The parenchyma of the juxtaoral organ in adult mice also showed epithelial cells positive for cytokeratin 14, a typical marker of the basal cells of the stratified epithelium. In adult mice, at the space between the parenchyma and the sheath, afferent nerve endings, elastic fibres, collagen fibres and sparse extracellular matrices were observed, similar to the findings reported for golden hamsters by Jeanneret-Gris [20]. These morphological structures may be involved in the transmission of mechanical stress from surrounding tissues to afferent nerve endings and may support the anterior–posterior-oriented structure.

Mérida-Velasco and colleagues [17] evaluated the development of the juxtaoral organ in rats, reporting that this anatomical structure is similar to the human juxtaoral organ. In rats, the juxtaoral organ develops from an epithelial condensation at the end of the transverse groove of the primitive mouth at embryonic day 14, then invaginates and disconnects from the oral epithelium (at embryonic day 15), forming a solid epithelial cord at embryonic day 16. At this stage, the juxtaoral organ presents three parts: anterior, middle, and posterior.

However, it is important to underline that, probably due to the lack of affordable methods to examine the distribution of the juxtaoral organ in experimental animals, there are very few studies to date on morphological, anatomical and functional juxtaoral organ aspects in species other than humans.

## 2. Juxtaoral Organ Anatomy

### 2.1. Gross Anatomy

The juxtaoral organ is a flat, tapered, solid strand of white tissue resembling a nerve and is not considered a macroscopically significant organ. It is generally described as 7–17 mm long and 1–2 mm wide [1,4,12,21]. In the cases in which the juxtaoral organ reaches 10 mm of diameter, this may be presumed to indicate a submucosal tumour or hyperplasia of the juxtaoral organ [10].

**Figure 1:**
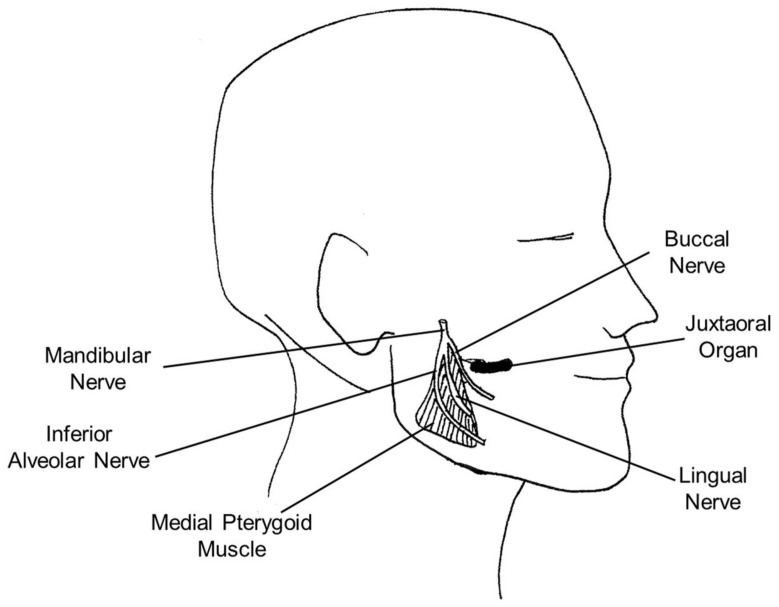
Schematic representation of human juxtaoral organ localization.

In our opinion, it is important to underline that, to date, there is no consistency of description of the anatomical localization of the juxtaoral organ, due to it usually being an incidental finding in biopsy specimens or resections and the evaluated cases being mainly from surgical resections or autopsy and not from analyses of targeted samples. However, it is prevalently described as lateral to oral cavity walls in the pterygomandibular space, as schematically depicted in Figure 1.

In detail, the juxtaoral organ was indicated as located within the soft tissue overlying the angle of the mandible in the spatium buccotemporale [8,14,21,22,23] and separated from the corpus adiposum buccae by the fascia buccotemporalis [12]. This organ was also described as in close proximity to the site used for inferior alveolar nerve block injection [24]. Other authors reported that the juxtaoral organ is located in the infratemporal region, at the level of the submucosa, in the retromolar trigone, medial to the medial pterygoid muscle and in relation with the pterygomandibular raphe [4,10,15]. Ramos-Vega and Roa [4] used, for the first time, head sections of a plastinated Asian male cadaver. Plastination is a preservation technique based on the replacement of tissue/body lipids and water by reactive polymers [25]. Ramos-Vega and Roa [4] performed this specific technique in order to identify correctly and faithfully the anatomical location and topographical relations of the juxtaoral organ in an adult subject. They thus confirmed that the anatomical localization of the juxtaoral organ is in the submucosa of the retromolar trigone in line with the pterygomandibular raphe, medial to the mandibular ramus, anteromedial to the medial pterygoid muscle and lateral to the base of the tongue. They also reported that the juxtaoral organ is closely related posteriorly with the lingual nerve and medially with the roots of the lower third molar. These observations could be used to assess the exposure and risk of injury to the juxtaoral organ during oral surgery.

Other studies also describe the juxtaoral organ as closely connected to the medial pterygoid muscle and innervated by the buccal nerve [1,3,26]. One study examined the juxtaoral organ in the posterior tongue [27]: Palazzolo and colleagues [27] described the juxtaoral organ as a neuroepithelial structure composed of non-keratinizing squamous epithelial nests associated with the subepithelial nerve plexus of taste buds. Furthermore, They speculated that the origin of the juxtaoral organ from embryologic structures was involved in tongue formation rather than originating from the epithelium of salivary glands, due to the lack of observation of any glandular or ductular differentiation. Notably, identification of the anatomical structure in the posterior tongue can prevent the misdiagnosis of cancer and prevent unnecessary overtreatment.

In an extensive literature review, Kennedy [2] reported that the most commonly described anatomical location of the juxtaoral organ is lingual to the mandible, but in keeping with published cases, it can also be found buccal to the mandible and in the posterior tongue.

### 2.2. Microscopical Anatomy

Understanding the morphology and microscopical anatomy is important in order to improve knowledge on the juxtaoral organ and thus help to identify its functional and diagnostic significance.

The juxtaoral organ consists of an epithelial parenchyma embedded in a highly organized connective tissue rich in nerves and sensory receptors [1,15,23].

The juxtaoral organ parenchyma presents non-keratinizing squamous epithelial cells organized in multilobulated and interconnected nests of different sizes and shapes, which may form pseudolumens [4,21,27]. These epithelial cells do not display mitotic figures and cytoplasmic clearing; brown pigmentation and dystrophic calcification may be observed occasionally [2,4].

The parenchyma of the juxtaoral organ also displays some cylindrical cells with clear cytoplasm-forming cords or glandular-like tubular structures [28]. The parenchyma is characterized by two types of cells: the first type is the more abundant and the cells have a diameter of 7–13 micron, whereas, the second type resemble dendritic cells [14,29]. In detail, the first cells resemble keratinocytes and have a cellular process including cytoplasmic inclusions, granules and large granular vesicles. These cells are linked to each other by tight junctions and desmosomes. The second cells have long processes that reach the first cells and present abundant endoplasmic reticulum. These second cells do not present desmosomes and are separated from the basal lamina through the interposition of the first type of parenchymal cells. The large granular vesicles of the first epithelial cells, together with the high enzymatic activity of all the parenchymal cells, suggested that the juxtaoral organ also features metabolic activity [1].

Some authors have demonstrated that the juxtaoral organ cell nests are immunohistochemically positive for vimentin, epithelial membrane antigen (weakly reactive), desmin, melan-A, a wide spectrum of cytokeratins and neuroendocrine markers (such as chromogranin, synaptophysin and neuron-specific enolase) [10,28,30]. The central epithelial cells are positive for cytokeratin 19 and the peripheral basal cells are positive for high molecular-weight keratin; however, no expression of S100 protein was observed [2]. The positivity for cytokeratins suggests that the juxtaoral organ epithelial nests share the immunohistochemical phenotype with non-keratinized stratified squamous cells [10,28]. Bahcelioglu et al. [16] reported positive immunoreactivity for the epidermal growth factor, transforming growth factor-alpha and nerve growth factor-beta at the parenchymal cell level of the juxtaoral organ in dogs. In particular, the authors observed strong and clear positivity of the parenchymal and capsular cells for transforming growth factor-alpha and nerve growth factor-beta, whereas the juxtaoral organ presents slightly positive immunoreactivity to the epidermal growth factor in the parenchymal cells (especially the central ones) and very strong positivity at the capsular cell level. The elevated enzymatic activity of the juxtaoral organ at the parenchymal cell level may potentially reflect a high metabolic activity of the cellular component. The close relationship between parenchyma and nerves, the granules positive for nerve growth factor-beta and the presence of neurosecretory-like granules within the cells, leads to the speculation of possible neurosecretory function(s) of the juxtaoral organ.

However, to date, immunohistochemistry studies of the juxtaoral organ are very limited.

The parenchyma of the juxtaoral organ is surrounded by connective tissue strata that originated from the stroma: the stratum fibrosum externum (or capsule) and the stratum fibrosum internum. The stratum externum is connected to the muscle fascia of the buccotemporalis [10]. The juxtaoral organ also presents a stratum nervosum featuring myelinated and unmyelinated nerve fibres and capillaries as well as a variety of slow- and fast-adapting sensory structures [1,12,15,23]. Inside these connective tissue strata there are mast cells, fibroblasts, lymphocytes and cells containing melanin pigment, as well as numerous capillaries, as well as collagenous microfibrils [1,16]. The stratum internum presents loose collagen fibrils and elastic microfibrils separated from the epithelial nests by a distinct basal lamina [1,10,16]. This inner layer has a thickness of about 50 nanometres.

Recently, Mérida-García et al. [3] performed a histomorphological study on juxtaoral organs obtained through the dissection of the infratemporal region in adult human cadavers. Using picrosirius red staining and immunohistochemistry, they evaluated the expression and arrangement of collagen type I and collagen type III. These analyses and the information obtained are important in improving knowledge on the morphology, structure and organization of the juxtaoral organ, which are then reflected in its properties and function(s). Interestingly, the authors reported that the externum fibrosum layer presents a predominance of type I collagen fibers arranged concentrically and that the internum fibrosum layer presents, conversely, a radial arrangement of type III collagen fibers. The type I collagen fibers give to the juxtaoral organ solidity and resistance to stretching and the type III collagen fibers provide it with important elastic properties. Suárez-Quintanilla and colleagues [14] noticed that the both internum and externum layers were strongly immunopositive for CD34, a known blood vessel marker [31], suggesting the presence of structures resembling capillaries.

**Figure 2:**
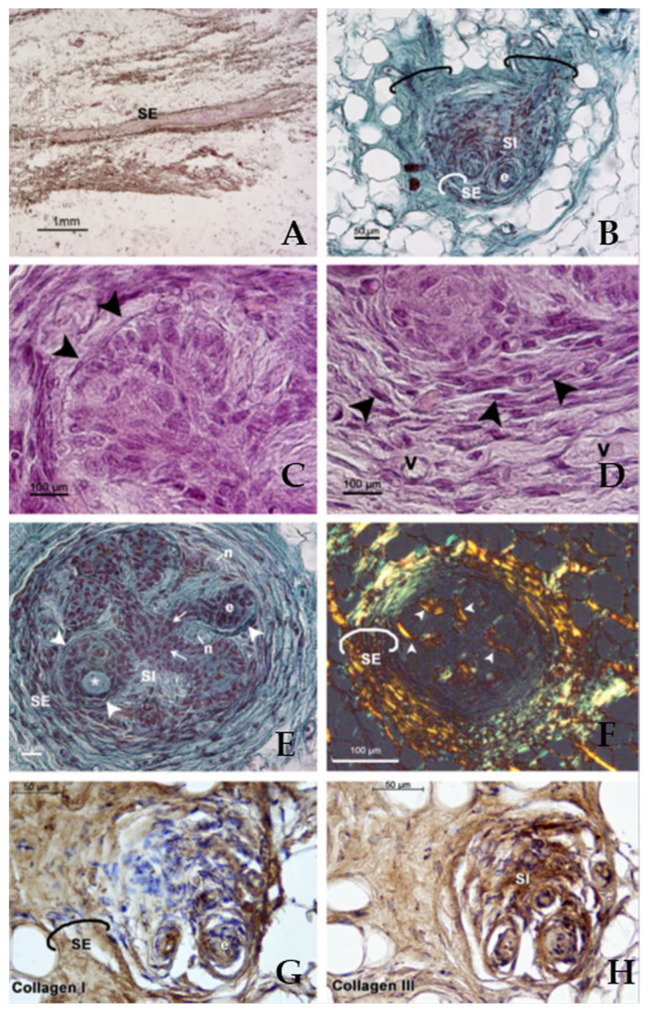
The morphology and microscopical anatomy of the juxtaoral organ. (**A**) Juxtaoral organ, longitudinal section. (**B**–**H**) Juxtaoral organ, frontal sections. (**C**) Epithelial nest. (**D**) Stratum fibrosum externum. (**E**) Nerve fibers in the stratum fibrosum internum in relation to continuity between epithelial nests (arrows). (**F**) Connective tissue strata surrounding the parenchyma of the juxtaoral organ showing that the collagen of the stratum fibrosum externum is arranged in concentric layers and the collagen of the stratum fibrosum internum is arranged radially (arrowheads). (**G**,**H**) Immunohistochemical expression of collagen type I (**G**) and of collagen type III (**H**). The white asterisk shows a vesicle with amorphous material. The white arrowheads indicate the basal membrane and the black arrowheads show the fibroblasts. The black line frames the bundle of dense connective tissue attaching the juxtaoral organ to the tissue surrounding it and the white line frames the stratum fibrosum externum. (e) Epithelial nest; (n): nerve fibers; (SE): stratum fibrosum externum; (SI): stratum fibrosum internum. (V): blood vessel. (**A**) Scale bar: 1 mm. (**B**,**G**,**H**) Scale bar: 50 μm. (**C**,**D**,**F**) Scale bar: 100 μm. (**E**) Scale bar: 20 μm. Reproduced with permission from Mérida-García et al., *Oral Diseases.* published by John Wiley and Sons., 2021. (License Number: 5211340000890).

The morphological features and microscopical anatomy of the juxtaoral organ can be observed in Figure 2.

## 3. Discussion

### Juxtaoral Organ: Clinical Relevance and Diagnostic Significance

In this section, we draw attention to the clinical importance and diagnostic implication(s) of the juxtaoral organ, emphasizing its considerable importance in clinical, surgical and pathological fields.

Numerous embryonic epithelial remnants are described in the oral region and, especially if intimately associated with peripheral nerves, can be a diagnostic and/or clinical pitfall. The juxtaoral organ is relevant in surgical practice due to its anatomical relationship with clinically significant adjacent structures (such as the lower third molar and lingual nerve). Furthermore, the juxtaoral organ being situated in the soft tissues of an anatomical area that is a common site of oral cancers and the fact that it presents epithelial nests, could lead to the false interpretation of an invasive carcinoma, leading to an unnecessary and extensive surgical procedure with a considerable impact on patient prognosis and quality of life [4,13,24,32,33]. The presence of lumen together with squamous-like cells may lead to confusion with salivary gland tumors or squamous carcinomas. In detail, squamous carcinoma would be the most likely mistaken identification of the juxtaoral organ; indeed, it has been reported that, based upon their typical association with nerve tissue, they can easily be confused with a perineural invasion of tumor cells [5,23,34]. In addition, the retromolar trigone is the preferred area for mucoepidermoid tumors and an extensive tumor excision in this area could wrongly include the juxtaoral organ with, again, significant impact on patient prognosis and quality of life [5]. The scientific literature contains various reports of the juxtaoral organ being wrongly identified and it is therefore fundamental to be aware of the existence of the juxtaoral organ in order to prevent diagnostic errors. Its clinical relevance is also related to the differential diagnosis with invasive processes of malignant neoplasia originating in the oral cavity. Oral pathology specialists must consider the morphology and anatomy of the juxtaoral organ, in order to avoid overdiagnosis and unnecessary overtreatment/removal [4], and subsequent therapy and prognosis, are strongly related to the correct diagnosis and thus the identification of the juxtaoral organ. The procedure of differential diagnosis, ensuring there is no misinterpretation and thus correctly identifying this organ, lies in the understanding of its anatomical, morphological and cytological features; clinical features are rarely helpful [4,5,14,22]. Kennedy [2], when identifying the standard for the correct juxtaoral organ diagnostic procedure, emphasized the fact that morphological and anatomical criteria, in contrast to clinical features, may help to differentiate between the juxtaoral organ and infiltrating carcinoma, as summarized in Table 1.

## 4. Conclusions

As previously reported, the juxtaoral organ is of considerable importance in clinical, surgical and pathological fields; in our opinion, further studies are needed to prevent misinterpretation and/or overtreatment.

Precise and in-depth anatomical knowledge is crucial to preventing the confusion of the juxtaoral organ with invasive carcinoma; thus, awareness of the juxtaoral organ is fundamental to avoiding diagnostic and clinical pitfall.

Despite various studies, no definitive conclusion has been reached and so, to date, additional evaluations are still needed to improve understanding of the function(s) linked to its anatomical features and thus to update current knowledge about the juxtaoral organ, adding new clinical perspectives.

## Figures and Tables

**Table 1:** The clinical relevance of the juxtaoral organ.

Juxtaoral Organ	Clinical Pitfall
Lumen and squamous-like cells	Salivary gland tumors Squamous carcinomas (most likely mistaken identification of the juxtaoral organ)
Lateral to oral cavity walls Into soft tissue of the retromolar trigone area	Oral cancers Mucoepidermoid tumors
Epithelial nests Association with nerve tissue	Oral cancers Perineural invasion of tumor cells
Glandular foci filled with colloid (rare)	Mucoepidermoid tumors

## Data Availability

Not applicable.
